# Comparison of volumetric and linear measurements of intestinal inflammation and treatment response in children with newly diagnosed ileal Crohn disease

**DOI:** 10.1007/s00330-025-11421-7

**Published:** 2025-02-12

**Authors:** Betul E. Derinkuyu, Andrew Bard, Iyad Naim, Jean A. Tkach, Lee A. Denson, Jonathan R. Dillman

**Affiliations:** 1 Department of Radiology, Cincinnati Children’s Hospital Medical Center, University of Cincinnati College of Medicine, Cincinnati, OH USA; 2 Motilent, London, UK; 3 Department of Pediatrics, Division of Gastroenterology, Hepatology, and Nutrition, University of Cincinnati College of Medicine, Cincinnati, OH USA

**Keywords:** Crohn disease, Magnetic resonance imaging, Segmentation, Volumetric measurement, Children

## Abstract

**Objectives:**

To compare the responsiveness of linear and volumetric assessments of intestinal inflammation in children with newly diagnosed ileal Crohn disease (CD) into treatment.

**Materials and methods:**

Twenty children with ileal CD (8 girls; mean age = 14.0 years) between May 2019 and April 2021 underwent research MRI at three time points—diagnosis, 6 weeks, and 6 months into treatment. For each examination, a radiologist measured the maximum wall thickness and length of disease. Then, the inflamed ileum was manually and semi-automatically segmented to measure the tissue volume. Mixed-effects models were used to assess the changes over time. Intra-class correlation was used to assess agreement between manual and semi-automated segmentation.

**Results:**

Length of disease decreased with medical treatment (19.2 vs. 12.2 vs. 8.0 cm; *p* = 0.002), 36% from baseline to 6 weeks and 58% from baseline to 6 months, while maximum bowel wall thickness also decreased over time (7.6 vs. 5.8 vs. 4.5 mm; *p* < 0.0001), 24% from baseline to 6 weeks and 41% from baseline to 6 months. Manual volumetric measurements demonstrated a significant treatment response (19.8 vs.11.6 vs.5.1 mL; *p* < 0.0001), 41% decrease from baseline to 6 weeks and 74% from baseline to 6 months. Using semi-automated segmentation, the volume decreased significantly over time as well (24.0 vs.15.1 vs.9.1 mL; *p* = 0.0007), 37% from baseline to 6 weeks and 62% from baseline to 6 months. There was good agreement between manual and semi-automated volumetric assessments (ICC = 0.78 [95% CI: 0.57–0.88]).

**Conclusion:**

Volumetric measurement of inflammation is responsive to medical treatment in CD and, as percentage compared to baseline, may show greater treatment response than linear measurements.

**Key Points:**

***Question*** *Volumetric measurements of intestinal inflammation related to Crohn disease may be superior to traditional linear measurements*.

***Findings*** *Volumetric measurements showed greater treatment response when compared to linear measurements, and there was good agreement between manual and semi-automated volumetric measurements of intestinal inflammation*.

***Clinical relevance*** *Volumetric assessment is a promising tool that may provide a novel biomarker for evaluating intestinal inflammation and treatment response in Crohn Disease*.

## Introduction

Crohn disease (CD) is a chronic autoinflammatory disorder affecting the gastrointestinal tract and is characterized by repeated episodes of transmural bowel wall active inflammation with variable intestinal damage over time [1, 2]. Magnetic resonance enterography and computed tomography enterography (CTE) are commonly used for both diagnosis and monitoring treatment response, including response to state-of-the-art biologic and small molecule therapies [2–4]. Typical imaging findings of bowel wall active inflammation include mural thickening and postcontrast hyperenhancement [5–7]. Linear measurements of bowel wall thickness and intestinal length of involvement are commonly performed to stage disease severity and extent, and changes in these measurements are commonly used to evaluate treatment response [5, 6]. Bowel wall thickness measurements can be subject to variability and depend on where the operator decides to measure along the course of the inflamed intestine. Similarly, length of disease can be challenging to measure on Picture Archiving and Communication Systems (PACS) as the inflamed bowel commonly courses in and out of plane.

Using purpose-built software, it is possible to segment inflamed bowel segments using either a manual, fully automated, or semi-automated approach [8–12]. It was found that semi-automatic techniques facilitate reproducible delineation of regions with active Crohn disease with good interobserver agreement for bowel wall thickness measurements [11]. To date, however, there is a paucity of data comparing linear measurements of intestinal active inflammation (i.e., bowel wall thickness, length of disease) to volumetric tissue assessments with the help of purpose-built software. It is conceivable that such a volumetric assessment of intestinal inflammation, which accounts for length of disease as well as bowel wall thickness along the entire length of the affected bowel segment, could provide a better overall estimate of inflammatory severity and extent as well as a more responsive biomarker to treatment response.

The purpose of this study was to compare the responsiveness (i.e., change over time) of conventional linear measurements and novel volumetric assessments of active intestinal inflammation in children with newly diagnosed ileal CD treated with anti-TNF medication. Second, we sought to compare the agreement between manual volumetric assessments of ileal inflammation and volumetric assessments obtained using a preclinical, semi-automated tool (GISeg software; Motilent).

## Methods

This prospective, single-center investigation was institutional review board-approved and compliant with the Health Insurance Portability and Accountability Act (HIPAA). Written informed consent as well as informed assent were obtained from all participants, as appropriate.

### Study sample

**Fig. 1:**
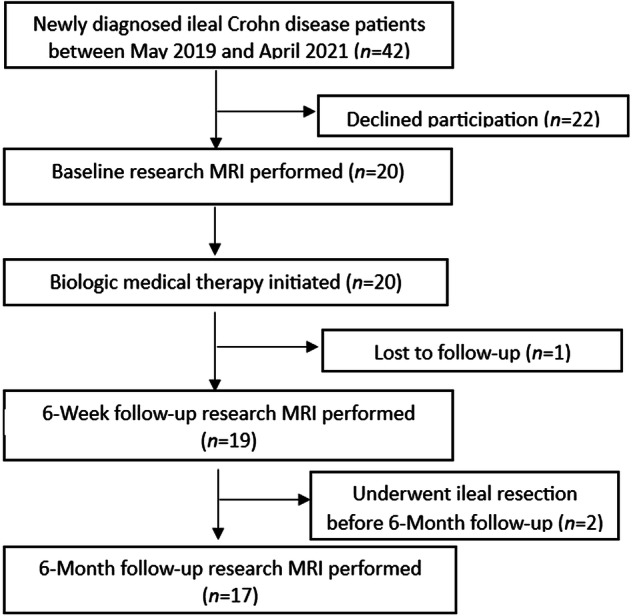
Flow diagram of patient selection

Between May 2019 and April 2021, we enrolled 20 children (≤ 18 years old) with newly diagnosed ileal CD, confirmed by ileocolonoscopy and MRI or CT, who were arranged to undergo anti-TNF-α medical therapy. Potential exclusion criteria included contraindication to MRI (e.g., claustrophobia or specific implanted medical devices), known or suspected pregnancy, and prior intestinal surgery with removal of the terminal ileum. Contemporaneous treatment with another non-anti-TNF-α anti-inflammatory medical treatment (e.g., immunomodulator or corticosteroid) was not an exclusion criterion. A participant flow diagram is presented in Fig. 1.

Participants in the current study have been included in multiple previous publications. These prior studies are unrelated to the current investigation and related to simplified magnetic resonance index of activity (sMaRIA) scoring of intestinal inflammation [13], quantitative assessment of mesenteric blood flow measurements using phase-contrast MRI [14], quantitative assessment of intestinal motility using cine MRI [15], and quantitative assessment of bowel wall T1 relaxation [16] in the setting of newly diagnosed pediatric small-bowel CD.

### Research MR imaging

All participants underwent dedicated research MRI examinations of the small bowel using a 1.5-T scanner (Ingenia, Philips Healthcare) at three time points—diagnosis (baseline) as well as 6 weeks (± 2 weeks) and 6 months (± 4 weeks) into medical treatment. The examinations were performed awake without sedation or anesthesia. In addition to a variety of quantitative pulse sequences that were performed and not included in the current investigation, anatomic imaging was carried out in all participants using coronal and axial T2-weighted SSFSE sequences (without fat saturation). SSFSE key pulse sequence parameters were as follows: section thickness, 5 mm; TE, 80 ms; FOV, 280 × 280 mm; matrix, 216 × 215; echo-train length, 112; parallel imaging (SENSE) acceleration factor, 2; and number of signals acquired, 1. All participants were asked to abstain from food and non-clear liquids for a minimum of 4 h before the research MRI examination. Participants drank 1000 mL of oral contrast material (Breeza, Beekley Medical), or a similar volume of water if the oral contrast material could not be tolerated, during the 45-min period before imaging. All participants successfully ingested the prescribed volume of oral contrast material or water. No intravenous contrast material or spasmolytic medication was administered as part of this research study.

### Image analyses

**Fig. 2:**
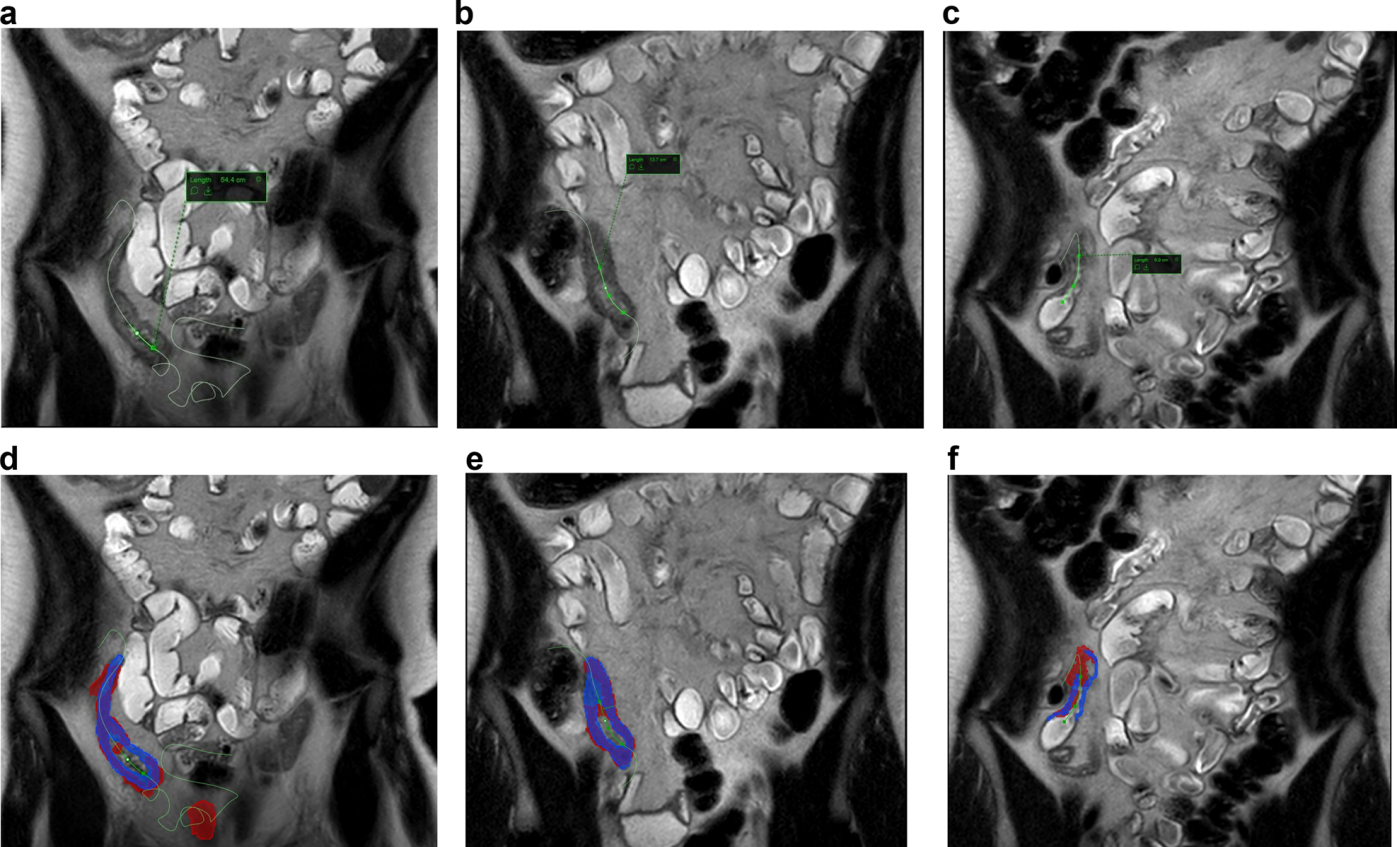
Eighteen-year-old male with newly diagnosed ileal Crohn disease. **a**–**c** Coronal single-shot fast spin-echo MR images at baseline (**a**), 6 weeks into medical treatment (**b**), and 6 months into medical treatment (**c**). Note the intraluminal curved green line demonstrating the length of disease called as centerline. The length of disease measured 54.4 cm (**a**), 13.7 cm (**b**), and 6.9 cm (**c**) at each time point, respectively. The manual segmentation is shown in blue color and semi-automated segmentation is shown in red color at baseline (**d**), 6 weeks into medical treatment (**e**), and 6 months into medical treatment (**f**)

For each research MRI examination and using coronal T2-weighted SSFSE images, a single investigator (B.E.D.) measured the maximum bowel wall thickness from the area of greatest ileal inflammation, under the supervision of a more experienced second investigator (both investigators were fellowship-trained pediatric radiologists). These measurements were placed perpendicular to the intestinal lumen and extended from the outer serosal to inner mucosal surfaces of the bowel wall. The length of the inflamed bowel segment was measured using a purpose-built tool that creates a linear measurement by placing multiple seed points along the affected intestinal lumen. This tool automatically interpolates a curved line between seed points, creating a so-called centerline that provides an exact length of disease (Fig. 2). Axial SSFSE images were used to corroborate the coronal SSFSE findings, including the determination of where ileal inflammation started and ended. All these measurements were made using GI segmentation software (Motilent).

**Fig. 3:**
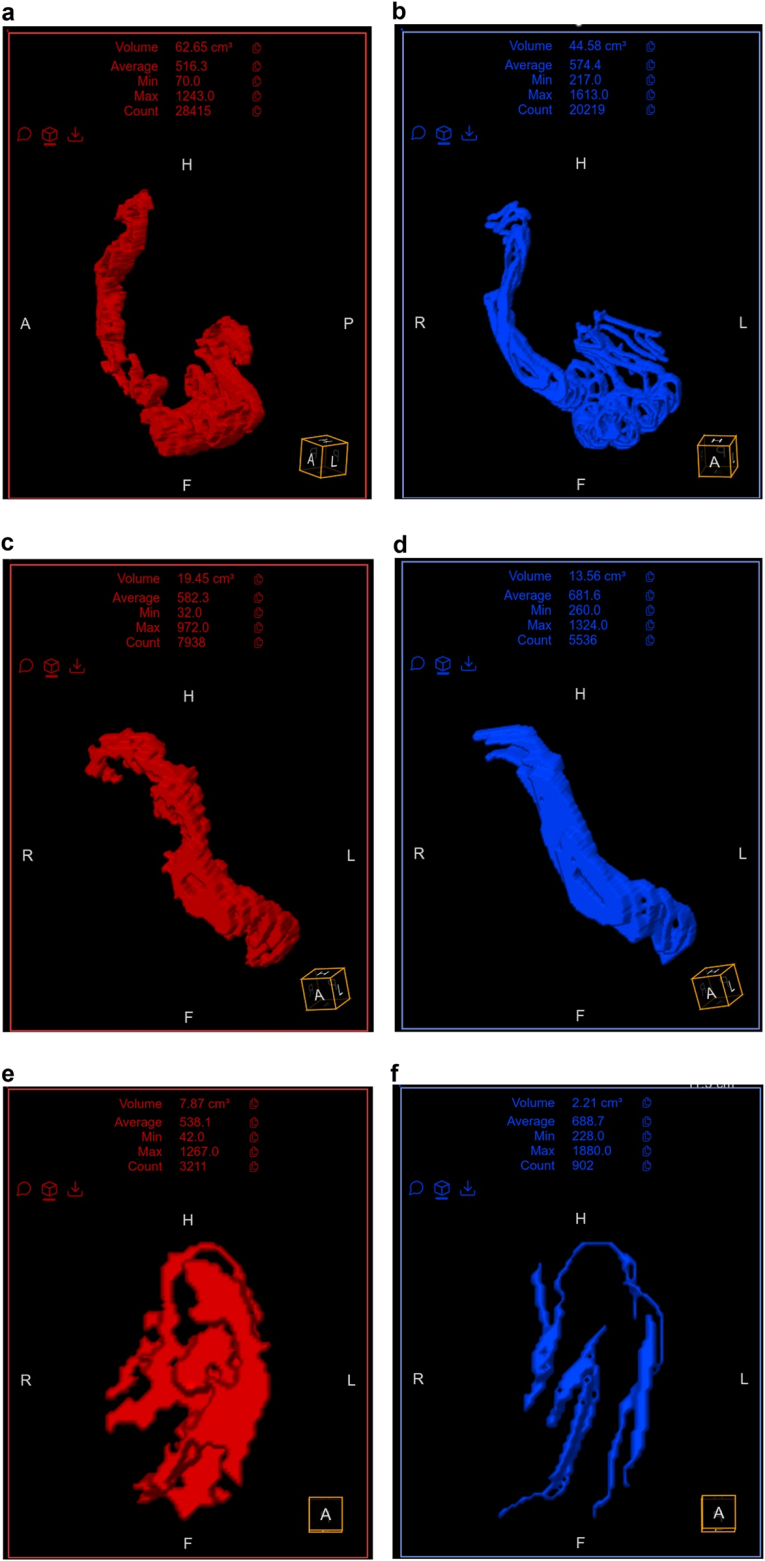
The 3D volumetric images of the same patient in Fig. 2 demonstrate the total volume of inflamed intestine. Based on semi-automated segmentations in red color, the volume of inflamed intestine measured 62.7 mL at baseline (**a**), 19.5 mL at 6 weeks into treatment (**c**), and 7.9 mL at 6 months into treatment (**e**). Based on manual segmentations in blue color, the volume of inflamed intestine measured 44.6 mL (**b**), 13.6 mL (**d**), and 2.2 mL (**f**) at each time point, respectively

Next, using the same software and research coronal T2-weighted SSFSE images, the inflamed ileal wall was manually segmented by the same operator along the length of the centerline generated above to create a volumetric assessment of intestinal inflammation (in mL) from each examination. These segmentations included only the bowel wall (from outer serosa to inner mucosa surfaces) and excluded the bowel lumen, including intraluminal contents. Finally, a novel software tool (Motilent) was used to generate semi-automated volumetric segmentations of inflamed ileum (Figs. 2, 3). Using the manually created centerline described above, this tool automatically segments the associated inflamed ileal wall by first using an unbiased k-means clustering technique to separate the image into contiguous partitions, then characterizing each partition according to a custom feature set and using these features as input to a random forest regressor which estimates the probability of each partition forming part of the final segmentation [17]. The mean bowel wall thickness was calculated from manual ileal segmentation masks using Distance Transform morphological operations [18]. This method processes the generated bowel wall mask to determine the thickness at each point along the mask’s length. The algorithm then averages these thickness values along all central pixels within the mask area, providing a mean thickness of the bowel wall for a given segment.

### Statistical analysis

Continuous data were summarized as means and standard deviations, whereas categorical data were summarized as counts and percentages. Mixed effects models for repeated measures data were used to assess if measurements changed over time in response to medical therapy, including maximum bowel wall thickness, length of disease, manual and semi-automated volumetric assessments of ileal inflammation, and mean bowel wall thickness. The mean percentage change between baseline to 6 weeks as well as baseline to 6 months was calculated for each measurement. Intra-class correlation was used to assess agreement between manual and semi-automated volumetric assessments, where agreement below 0.50 was poor; agreement between 0.50 and 0.75 was moderate; agreement between 0.75 and 0.90 was good; and agreement above 0.90 was excellent [19]. Finally, Pearson correlation (*r*) was used to evaluate the relationships between the different types of measures, with measurements from all three time points combined for these analyses.

A value of *p*-value less than 0.05 was considered statistically significant for all inference testing; 95% confidence intervals were calculated as appropriate. Statistical analyses were performed using GraphPad Prism software (version 9.5.0 for Windows, GraphPad Software) and MedCalc statistical software (version 20.111).

## Results

### Study sample

We enrolled 20 pediatric participants (8 girls and 12 boys) with newly diagnosed CD and ileal involvement during the study period. The mean age was 14.0 years (range, 12–18 years). A single participant had only baseline imaging and was lost to follow-up due to the COVID-19 pandemic. Two participants did not undergo follow-up research imaging at 6 months into treatment due to surgical ileal resection between the 6-week and 6-month study visits. These three patients with missing data were included in statistical analyses using available data.

### Linear measurements

**Fig. 4:**
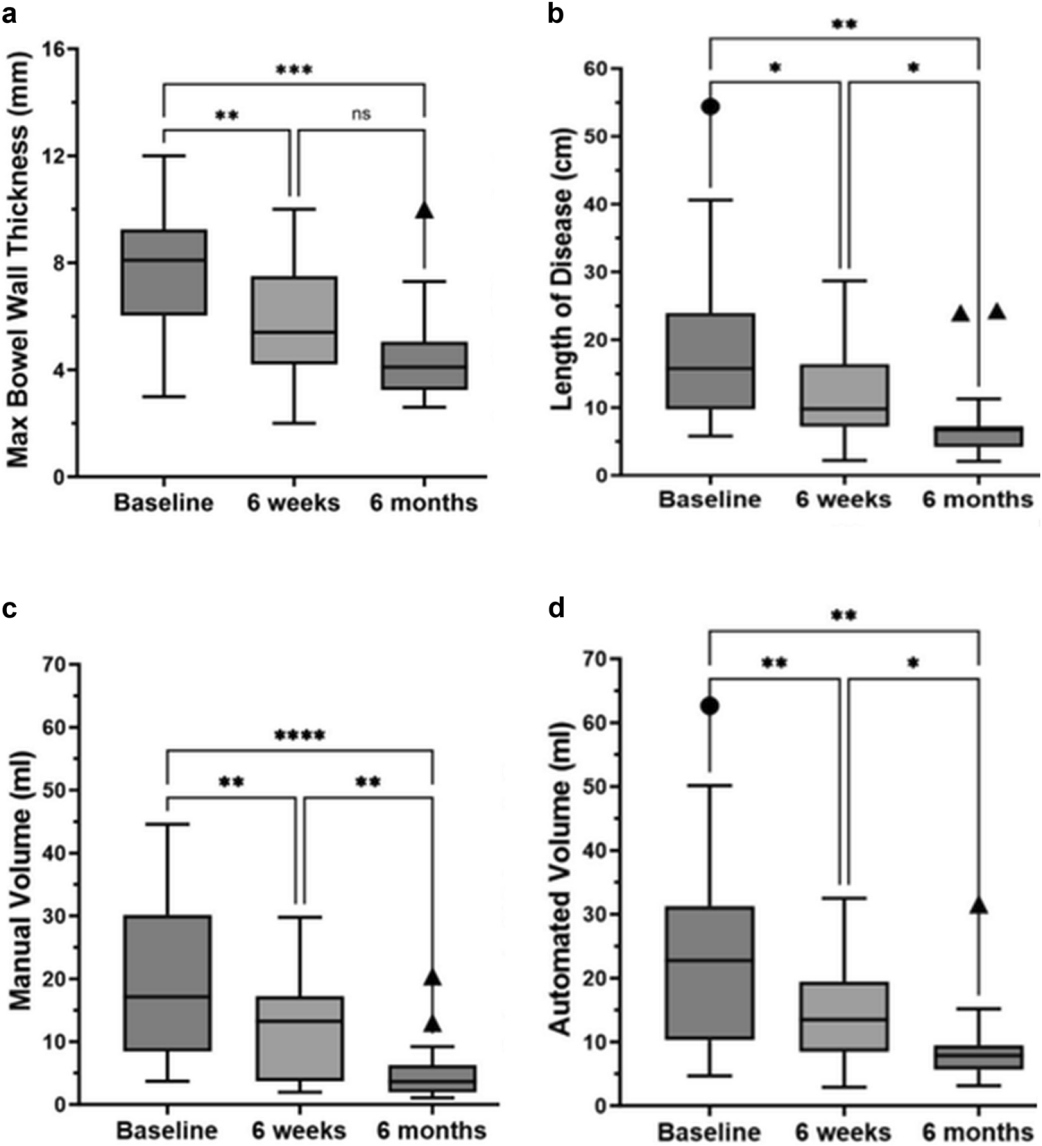
Tukey box plots showing the change over time in measurements of maximum bowel wall thickness (mm) (**a**), length of disease (cm) (**b**), manual volumetric assessment (mL) (**c**), and semi-automated volumetric assessment (mL) (**d**) in patients with newly diagnosed ileal Crohn disease. Boxes extend from the first through the third quartiles of datasets, horizontal lines in boxes represent median values, and whiskers represent 1.5 x IQR (or minimum and maximum values in the absence of statistical outliers). Circles and triangles represent statistical outliers. (* < 0.05, ** < 0.01, *** < 0.001, **** < 0.00001)

There was a significant decrease in maximum bowel wall thickness over time (baseline vs. 6 weeks vs. 6 months: 7.6 ± 2.1 vs. 5.8 ± 2.2 vs. 4.5 ± 1.9 mm; *p* < 0.0001). This maximum bowel wall thickness measurement decreased 24% from baseline to 6-week and 41% from baseline to 6-month examinations (Fig. 4).

There was also a significant decrease in length of disease based on centerline measurements over time (baseline vs. 6 weeks vs. 6 months: 19.2 ± 12.8 vs. 12.2 ± 7.3 vs. 8.0 ± 6.4 cm; *p* = 0.002). This measurement decreased 36% from baseline to 6 weeks and 58% from baseline to 6 months examinations (Fig. 4).

### Volumetric measurements

Based on manual segmentations, the volume of inflamed ileum decreased in response to medical therapy. Specifically, mean volume of disease was 19.8 ± 13.0 mL at baseline, 11.6 ± 8.0 mL at 6 weeks into treatment, and 5.1 ± 5.0 mL at 6 months into treatment (*p* < 0.0001). Manual volumetric measurements decreased 41% between baseline to 6 weeks and 74% between baseline to 6 months (Fig. 4).

Based on semi-automated segmentations, the volume of inflamed ileum also decreased in response to medical therapy. Specifically, mean volume of disease was 24.0 ± 15.4 mL at baseline, 15.1 ± 8.7 mL at 6 weeks, and 9.1 ± 6.6 mL at 6 months into treatment (*p* = 0.0007). Sem-automated volumetric assessments decreased 37% between baseline to 6 weeks and 62% between baseline to 6 months (Fig. 4).

There was good agreement between manual and semi-automated volumetric measurements of intestinal inflammation (ICC = 0.78 [95% CI: 0.57–0.88]).

Finally, using manual segmentations, mean bowel wall thickness was measured along the entire length of disease in a semi-automated manner. Mean bowel wall thickness was 3.6 ± 1.0 mm at baseline, 3.3 ± 1.2 mm at 6 weeks, and 2.6 ± 0.9 mm at 6 months (*p* = 0.01). This measurement decreased 8% between baseline to 6 weeks and 28% between baseline to 6 months.

### Correlation between different measurement types

Correlations between the different measurement types are presented in Table 1.

**Table 1:** Associations between different measurement types using Pearson correlation

	Maximum bowel wall thickness	Length of disease	Manual disease volume	Automated disease volume
Maximum bowel wall thickness	-	-	-	-
Length of disease	−0.02 (−0.28 to 0.24) [0.88]	-	-	-
Manual disease volume	0.49 (0.26–0.67) [< 0.0001]	0.65 (0.46–0.78) [< 0.0001]	-	
Automated disease volume	0.06 (−0.21 to 0.31) [0.68]	0.94 (0.89–0.96) [< 0.0001]	0.69 (0.52–0.81) [< 0.0001]	-

95% confidence intervals are in parentheses, while *p*-values are in brackets

## Discussion

In the current study, we found that both conventional linear measurements of intestinal inflammation, including maximum bowel wall thickness and length of disease, as well as our novel volumetric assessments of disease activity, decrease over time in response to anti-TNF-α medical therapy. Interestingly, volumetric assessments based on manual segmentations demonstrated the greatest change as a percentage of baseline at both 6-week and 6-month follow-up imaging. This observation raises the possibility that volume-based measures of tissue inflammation may be more responsive to treatment effects than conventional linear measurements in patients with ileal CD. Importantly, manual measurements of disease volume only moderately correlated with bowel wall thickness and length of disease (*r* = 0.49–0.65), again suggesting that volumetric assessments may provide a unique tool for disease evaluation. However, novel cutout values of responsiveness for volumetric assessment will need to be established with large samples to validate that the greater percentage means the greater therapy response.

Volumetric assessments of intestinal inflammation based on semi-automated segmentation of the bowel also decreased significantly in response to treatment and showed a degree of responsiveness approaching that of manual volumetric assessments and greater than that of linear measurements. Interestingly, these semi-automated measurements of disease volume highly correlated with length of disease but failed to correlate with bowel wall thickness, unlike manual volumetric measurements, which moderately correlated with both bowel wall thickness and disease length. Importantly, these semi-automated measurements, which require a human operator to place a centerline along the length of the inflamed bowel, showed good statistical agreement with manual volumetric assessments. Using a 3D residual convolutional neural network, Lamash et al developed a semi-automated algorithm for segmenting the bowel wall and lumen using fat-suppressed postcontrast T1-weighted MR images [8, 20]. Using CTE data, Stidham et al also developed a semi-automated algorithm for segmenting the bowel wall and lumen by iteratively modeling a polynomial grid on the transition edges between wall to mesentery and lumen to wall using super-pixel voxel segmentation followed by k-means classification [12]. Both tools require a human operator to place reference or seed points in the bowel lumen, similar to our study.

Our bowel segmentation tool, as well as other previously described semi-automated methods for segmenting the bowel, has the potential to offer novel biomarkers for disease assessment that can likely be incorporated into clinical practice. However, for such tools to be adopted, we believe the generated segmentations must be both accurate and easily edited with minimal additional human effort when refinement is deemed necessary. The manual segmentation pipeline in our study required about 10 to 20 min per examination and varied based on the length of disease, whereas the semi-automated segmentation required considerably less operator time as it only required placement of a centerline within the inflamed ileum.

Volumetric assessments of bowel wall inflammation may have multiple advantages over conventional linear measurements. First, volumetric measurements likely provide a more global assessment than either bowel wall thickening or length of disease alone, as tissue volume incorporates both features. Individual measures of bowel wall thickness fail to account for overall mean bowel wall thickness and variability in thickness along the length of the diseased segment. Furthermore, length of disease fails to account for bowel wall thickness. Second, it is conceivable that volumetric assessments may demonstrate superior inter-radiologist agreement, particularly when compared to measurements of bowel wall thickness. This is because the mural thickness of an inflamed bowel segment can vary considerably along its length and the exact location where the thickness is measured is subject to operator-related variability. Finally, volumetric segmentations of the inflamed intestine can be used to generate other potential biomarkers of disease, including mean bowel wall thickness (as opposed to manual measurement of maximum bowel wall thickness) as presented in the current study. Interestingly, this particular measurement was the least responsive of any assessment performed, again perhaps due to variability in mural thickness along the length of inflamed bowel segments and the contribution of areas showing only mild wall thickening.

Our study has limitations. First, our study sample is relatively small, including only 20 pediatric patients with newly diagnosed ileal CD. Second, all the linear measurements and manual volumetric assessments were performed by a single investigator, under the supervision of a more experienced second investigator (both investigators were fellowship-trained pediatric radiologists). Thus, the interobserver agreement of these different linear and volumetric measurements was unable to be assessed. But the volumetric measurements were in good agreement in intra-class correlation between manual and semi-automated measurements. Third, our study assessed the responsiveness of linear and volumetric measurements in the setting of ileal CD. We did not evaluate how these measurements performed in other areas of inflamed bowel, such as the colon. Finally, all measurements were performed using a single software platform, although there is no reason to expect different results from other platforms that can make comparable measurements.

In conclusion, both linear and volumetric measurements of ileal inflammation are responsive to medical treatment, changing significantly over time. As a percentage compared to baseline, manual volumetric assessments may show a greater treatment response than linear measurements and may provide a novel biomarker for evaluating intestinal inflammation, thereby assisting radiologists in reporting objective measures of disease status as well as empowering clinical decision-making. Further investigations are needed to determine the clinical value of volumetric versus linear measurements and how such changes in these assessments correlate with endoscopy, histology, and clinical outcomes.

## Abbreviations

CTE: Computed tomography enterography
CD: Crohn disease
